# Preferences for Using a Mobile App in Sickle Cell Disease Self-management: Descriptive Qualitative Study

**DOI:** 10.2196/28678

**Published:** 2021-11-30

**Authors:** Tilicia L Mayo-Gamble, Delores Quasie-Woode, Jennifer Cunningham-Erves, Margo Rollins, David Schlundt, Kemberlee Bonnet, Velma McBride Murry

**Affiliations:** 1 Department of Health Policy and Community Health Jiann-Ping Hsu College of Public Health Georgia Southern University Statesboro, GA United States; 2 Center for Disease Control and Prevention Foundation Atlanta, GA United States; 3 Department of Internal Medicine Meharry Medical College Nashville, TN United States; 4 Department of Pediatrics Aflac Cancer and Blood Disorders Center Emory University School of Medicine Atlanta, GA United States; 5 Department of Psychological Sciences College of Arts and Sciences Vanderbilt University Nashville, TN United States; 6 Department of Health Policy Vanderbilt University School of Medicine Nashville, TN United States; 7 Department of Human and Organizational Development Peabody College Vanderbilt University Nashville, TN United States

**Keywords:** sickle cell disease, digital technology, rural, mHealth app, patient-centered technology, mobile health, health outcomes, hematology, mobile phone

## Abstract

**Background:**

Individuals with sickle cell disease (SCD) and their caregivers may benefit from technology-based resources to improve disease self-management.

**Objective:**

This study explores the preferences regarding a mobile health (mHealth) app to facilitate self-management in adults with SCD and their caregivers living in urban and rural communities.

**Methods:**

Five community listening sessions were conducted in 2 urban and rural communities among adults with SCD and their caregivers (N=43). Each session comprised 4 to 15 participants. Participants were asked questions on methods of finding information about SCD self-care, satisfaction with current methods for finding SCD management information, support for SCD management, important features for development of an mHealth app, and areas of benefit for using an mHealth app for SCD self-management. An inductive-deductive content analysis approach was implemented to identify the critical themes.

**Results:**

Seven critical themes emerged, including the current methods for receiving self-management information, desired information, recommendations for communicating sickle cell self-management information, challenges of disease management, types of support received for disease management, barriers to and facilitators of using an mHealth app, and feature preferences for an mHealth app. In addition, we found that the participants were receptive to using mHealth apps in SCD self-management.

**Conclusions:**

This study expands our knowledge on the use of mHealth technology to reduce information access barriers pertaining to SCD. The findings can be used to develop a patient-centered, user-friendly mHealth app to facilitate disease self-management, thus increasing access to resources for families of patients with SCD residing in rural communities.

## Introduction

### Background

Successful disease self-management improves health outcomes and decreases overall health care use [1,2]. For patients with chronic conditions, who are living in rural or medically underserved communities, self-management is essential because of their limited access to health care services and specialty care [3]. Technological advances present an opportunity for patients to access resources and health care providers to facilitate disease self-management. Past research has demonstrated that technology-based self-management tools are effective platforms for improving communication with health care providers, for patient education, for goal-setting and self-monitoring, for health care delivery, and for patient engagement [4-6]. Furthermore, health care organizations promote the use of technology-based interventions to minimize the disparities among vulnerable populations and extend the reach of care [7-12]. Technology-based interventions could potentially improve self-management in individuals with sickle cell disease (SCD), particularly for those residing in rural areas with limited access to specialty care (eg, hematology).

SCD is a blood disorder caused by the inheritance of 2 abnormal beta-globin alleles [13]. The most common form of SCD, both in the United States and worldwide, is homozygous hemoglobin SS, SCD/sickle cell anemia [14]. The disease affects approximately 100,000 individuals in the United States [15-17]. Individuals with SCD may experience acute and chronic complications because of ongoing vaso-occlusion crises secondary to hypoxia-induced sickling [18]. Acute pain is a hallmark of vaso-occlusion crises [19]. However, these individuals can develop stroke, priapism, and acute chest syndrome. Over time, acute vaso-occlusion episodes can result in chronic disease manifestations in end organs, such as poor renal, cardiac, pulmonary, and vascular dysfunction [20]. Therefore, disease management with a multidisciplinary team is crucial for managing SCD.

Although disease management is important, access to subspecialists is not readily available to all patients with SCD. Furthermore, education and interactive methods that prepare patients with SCD to implement self-care strategies to manage their disease are also limited. Therefore, having access to self-management tools could lead to a better understanding of disease management, increased adherence to self-care behaviors, and enhanced management of physical symptoms among individuals with SCD. Evidence of effective SCD self-management includes medication adherence, body temperature control, attention to appropriate food intake and hydration, and moderate exercising [21,22].

### Objectives

Self-management programs incorporating mobile health (mHealth) apps are emerging and have demonstrated success in self-care behaviors (eg, tracking pain, communicating with providers, and communicating with other individuals with SCD) in research studies targeting adolescents and young adults with SCD [23-25]. However, many of these studies did not include adults beyond the age of 22 years, who could also benefit from the mHealth resources. Furthermore, to our knowledge, there are no studies that have elicited input from patients on the usefulness of SCD mHealth apps for self-management, with a noticeable omission of patients and caregivers in rural communities [26]. These research gaps limit our insight into how mHealth apps could improve self-management among patients with SCD and caregivers, in general, and those residing in rural areas with limited access to subspecialty care, in particular. Tailoring health management technology to align with all needs of patients with SCD and preferences is necessary to increase access but may have implications for acceptance of these programs [27]. This study aims to contribute to the research and disease self-management practices of adults with SCD and their caregivers. Our sample included participants residing in urban and rural communities. The overall purpose of our study was to describe the preferences for mHealth app features designed to facilitate disease self-management.

## Methods

### Partnership

The researchers collaborated with the Sickle Cell Foundation of Tennessee (SCFT), a community-based organization serving patients with SCD and their caregivers. For this study, caregivers included the parents and spouses of individuals with SCD [28-30]. Representatives of the foundation were equal partners throughout each phase of the study, as described in the subsequent sections.

### Recruitment and Study Population

The participants were recruited through the SCFT. Recruitment strategies included sending emails through the SCFT listserv and announcements made through SCFT support group meetings. Flyers were also distributed at hematology clinics in Memphis and Nashville, Tennessee. When participants contacted the organization or the researchers to express interest, they were asked about their city of residence. This self-reported information allowed us to ensure geographic diversity in the sample by including representation for participants from both urban and rural communities.

Five community listening sessions were conducted with adults having SCD and their caregivers in urban (3/5, 60%) and rural communities (2/5, 40%). A community listening session is a qualitative research method in which a group of people with diverse backgrounds and perspectives express their views on an important issue [31]. These sessions have demonstrated success because they are led by a trained community leader who is well-known within the target group and are less structured than focus groups [31,32]. Our sessions ranged from 4 to 15 participants each (N=43).

### Training

Before the community listening sessions, the research team conducted facilitator training using a manualized moderator’s guide. Facilitators included the SCFT director and 2 community health workers who were well-known to the Tennessee SCD community through their advocacy. The manualized moderator’s guide was developed jointly by the research team and the SCFT community partners. Members of the research team were PhD-level researchers with a qualitative research experience. In addition to receiving training in SCD management, the community listening session facilitators were trained in qualitative research as part of a larger patient engagement project [33]. Another aspect of the training included a 2-hour session on practical group engagement, which consisted of dyadic role playing, where the facilitators engaged in reflective techniques to encourage group discussion and establish group cohesion.

The questions posed during the community listening session focused on (1) methods to find information about SCD self-care, (2) satisfaction with current methods for finding SCD management information, (3) support for SCD management, (4) important features of an mHealth app, and (5) benefits of using an mHealth app for SCD self-management.

### Procedures

Written informed consent was obtained from all the participants. The community listening sessions lasted approximately 2 hours and were audio-recorded. The facilitators explained the *ground rules*, which included the following statements: (1) everyone is encouraged to participate, (2) be respectful of other participants’ responses, (3) there are no wrong or right responses, (4) one participant should respond at a time, and (5) feel free to ask questions anytime during the session. Before beginning the session, the participants completed a brief survey assessing their demographics and technology access. Each participant was reimbursed with US $25 for partaking in the study. This study was approved by the institutional review board of Vanderbilt University.

### Analysis

Coding and analysis were managed by the Vanderbilt University Qualitative Research Core, led by a PhD-level psychologist. Before the analysis, all sessions were commercially transcribed verbatim. An inductive-deductive content analysis approach was implemented to identify the critical themes [34]. The consolidated criteria for reporting qualitative research, an evidence-based qualitative methodology checklist, was used to guide the coding and analysis of the data [35]. Study questions and a preliminary review of transcripts were used as guides for developing a hierarchical coding system. A preliminary review of transcripts began after 2 listening sessions, allowing experienced coders to ensure reliability in using the coding system. Reliability is a process in which coders establish an agreement upon understanding the coding system [36]. Two coders encoded the same transcript and then compared and discussed any differences between their codes. The finalized code was used as data. After reaching consensus on the 2 transcripts, the subsequent transcripts were independently coded [36]. The coding of each transcript was compared, and any discrepancies were resolved to create a single-coded transcript. Each sentence was treated as a separate quote and assigned to 5 different codes. The transcripts were then combined and sorted using code. The analysis consisted of interpreting the coded quotes and identifying higher-order themes using an iterative inductive-deductive approach.

## Results

### Participant Characteristics

Table 1 presents the demographic characteristics of the participants. Most of the participants were women (30/43, 70%) with an average age of 45 years (SD 12.3), had an SCD genotype SS or SC (19/43, 44%), indicated their health status as fair to good (25/43, 58%), and had a household income of <US $35,000 (25/43, 58%). Nearly half of the study participants (20/43, 47%) were unemployed. All the participants owned a smartphone to access mobile technology. Most participants had a smartphone with an iPhone (Apple Inc) operating system (25/43, 58%) and felt comfortable connecting to the internet on their smartphone (20/43, 47%). There were 67% (29/43) of participants who agreed that if an mHealth app offered information on SCD self-management, they would use it.

**Table 1:** Characteristics of patients and caregivers (N=43).

Characteristics		Values
Age (years), mean (SD)		44.8 (SD 12.3)
**Sex, n (%)**		
	Female	30 (70)
	Male	13 (30)
**SCD^a^ genotype, n (%)**		
	Hb^b^ SS^c^	10 (23)
	Hb SC^d^	9 (21)
	Hb Sβ0^e^	1 (2)
	Hb Sβ+^f^	4 (9)
	Unknown	4 (9)
**Stakeholder group,** **n (%)**		
	Patient	36 (84)
	Caregiver	7 (16)
**Health insurance status,** **n (%)**		
	Uninsured	6 (14)
	Private insurance	13 (30)
	Medicaid or Medicare	19 (44)
	Unknown	5 (12)
**Perceived health status,** **n (%)**		
	Excellent	4 (9)
	Very good	9 (21)
	Good	10 (23)
	Fair	15 (35)
	Poor	1 (2)
	Unknown	4 (9)
**Education,** **n (%)**		
	Some HS^g^	3 (7)
	High school diploma or GED^h^	4 (9)
	Some college	17 (40)
	College degree	13 (30)
	Unknown	6 (14)
**Marital status,** **n (%)**		
	Single	23 (54)
	Married	10 (23)
	Divorced	6 (14)
	Unknown	4 (9)
**Employment status,** **n (%)**		
	Unemployed	20 (47)
	Employed (part time)	7 (16)
	Employed (full time)	12 (28)
	Unknown	4 (9)
**Household income level (US $),** **n (%)**		
	≤10,000	8 (19)
	10,001-25,000	12 (28)
	25,001-35,000	5 (12)
	35,001-45,000	4 (9)
	>45,000	9 (21)
	Unknown	5 (12)

^a^SCD: sickle cell disease.

^b^Hb: hemoglobin.

^c^Hb SS: sickle cell disease or sickle cell anemia.

^d^Hb SC: sickle cell C disease.

^e^Hb Sβ0: sickle cell beta thalassemia zero.

^f^Hb Sβ+: sickle cell beta thalassemia plus.

^g^HS: high school.

^h^GED: general educational development.

In capturing the participants’ perspectives, 7 themes emerged: (1) current methods for receiving self-management information; (2) desired information; (3) recommendations for communicating sickle cell self-management information; (4) challenges of disease management; (5) types of support received for disease management; (6) barriers to and facilitators of using an mHealth app; and (7) feature preferences for an mHealth app. For each major theme, 2 or more categories were identified and have been presented in Textbox 1 along with illustrative quotes.

**Current methods for receiving self-management information. Illustrative quotes are examples from community listening participants used to develop the categories.**

**Illustrative quotes**

**Health care providers:**

- “The doctor is going to tell to take my medication.” [LS1]
- “It’s through your doctors, is the main thing.” [LS1]
- “Social media, the doctor.” [LS2]

**Personal experience:**

- “I've been going through it for so many years I kind of know what the doctors gonna tell me.” [LS1]
- “The years of experience having it helps you to control it better.” [LS2]
- “I've been going through this all my life so I know a pretty bit a much about it.” [LS3]
- “A lot of learned things that we can, and can’t, or shouldn’t do through trial and error.” [LS4]

**Internet:**

- “If you’re not able to be with your doctor at that time where you can find something.” [LS1]
- “I get a lot from social media.” [LS2]
- “On the internet.” [LS3]
- “We used the websites in early days.” [LS4]

**Other individuals with sickle cell disease:**

- “Getting our men to talk together.” [LS2]
- “Talking to each other, it’s like we’re the same people sometimes because we all go through the same thing, and we bounce information off of one another.” [LS4]
- “My grandmother already had it, so we already understood how we was going to get through this.” [LS4]

### Theme 1: Current Methods for Receiving Self-management Information

This theme explained how the participants learned about the various methods of disease management. We identified 4 categories of these methods: health care providers, personal experience, internet, and other individuals with SCD. Health care providers were the primary source of self-management information. The participants admitted to becoming more knowledgeable about SCD and managing its symptoms through their health care providers, “The doctor is going to tell to take my medication” (LS1). However, because every patient is different, participants also indicated that they relied on their personal lived experiences to guide self-management. For example, one participant stated, “The years of experience having it helps you to control it better” [LS2]. Participants also mentioned learning about sickle cell (disease) from other individuals with SCD. This appears to be an important method for information that resonates among participants; “Talking to each other, it’s like we’re the same people sometimes because we all go through the same thing” [LS4]. In addition, participants stated that the internet was a source of learning about self-management techniques. Specific internet sites included finding information on social media. One participant stated, “I got a lot from social media” [LS2].

### Theme 2: Desired Information

The *desired information* theme reflected the areas in which the participants wanted more knowledge (Textbox 2). The 2 categories of information were SCD and SCD treatment. Participants expressed a desire to learn more about the differences in symptoms among males and females, along with steps to manage their care: “I would recommend something along the lines of distinguishing protocols and forces of action for male and female” [LS1]. Some other participants had questions about disease management. For example, one participant indicated, “Do’s and don’ts. Yeah, just some basic lists, create a list of those types of things to share and communicate” [LS4]. Overall, despite receiving care for SCD, knowledge gaps still existed between the disease and the options to facilitate its self-management.

**Desired information. Illustrative quotes are examples from community listening participants recruited to develop the categories.**

**Illustrative quotes**

**Sickle cell disease (eg, types and symptoms):**

- “Any other stress factors or different things like that, that I think are different between men and women.” [LS1]
- “I can concur there is a big dip there (surrounding SCD) for the college students struggling to figure out.” [LS2]
- “Direct sickle cell. Like you said it’s different ones. The ones pertaining to yourself.” [LS3]
- “I just wonder why it’s not out there a bit more about the trait that has symptoms.” [LS3]

**Treatment:**

- “The biggest gap that I have seen in coming from the standpoint of the patient, is a transitional period.” [LS2]
- “In Memphis about treatment about possible cure for sickle cell disease.” [LS3]
- “New studies, new medications.” [LS4]
- “I think that it’s kind of going back to that point where I think about some of the cancer examples.” [LS5]
- “I’ve heard about where they’re super engaged. They know about this study being done over there.” [LS5]

### Theme 3: Recommendations for Communicating Sickle Cell Self-management Information

This theme describes the participants’ reflections on strategies to increase their knowledge of self-management (Textbox 3). The categories within this theme included suggestions on how the information could be improved, how the information was used in daily disease management, and the preferred methods for receiving the information. The participants agreed that communicating information within a community was important. For example, one participant stated, “I think when you go back out to these communities there should be probably a stronger dialogue between where are we in terms of what can we do and what they want to see done” [LS5]. Regarding the use of information for daily disease management, a participant stated, “We have to kind of construct the feedback you get from the general population in a way that we can act on” [LS5]. For the preferred methods for receiving information, the participants discussed tailoring of information. For example, “I think that part of the trick is finding out how those are incorporated into how a person goes through their life” [LS5]. Both categories seemed to indicate a need for more specific information that may be incorporated into daily disease self-management. Although many discussed using the internet to receive information, one participant also acknowledged the importance of finding reputable sites, “They taught you to actually go to reputable sites and look for stuff because we know that anybody can put stuff on the internet” [LS2].

**Recommendations for communicating sickle cell self-management information. Illustrative quotes are examples from community listening participants recruited to develop the categories.**

**Illustrative quotes**

**How information could be improved:**

- “The biggest gap that I’ve seen in coming from the standpoint of the patient, is a transitional period. It’s either pediatrics or they’re adults.” [LS2]
- “You’re right. Stop telling these kids they not going to live long.” [LS4]
- “I think when you go back out to these communities there should be probably a stronger dialogue between where are we in terms of what can we do and what they want to see done.” [LS5]

**How information is used for daily disease management:**

- “And they can email me that list or it could show up somewhere for me to access it.” [LS1]
- “We have to kind of construct the feedback you get from the general population in a way that we can act on.” [LS5]

**Preferred methods for receiving information:**

- “And it would be in my phone so the next time I go, and be like, you know, you see this, you know.” [LS1]
- “Put it on the phones so I can get a reminder. Put on the calendar, whatever.” [LS4]
- “I think that part of the trick is finding out how those are incorporated into how a person goes through their life.” [LS5]

### Theme 4: Challenges of Disease Management

This theme captures the difficulties experienced by the participants in maintaining a healthy lifestyle with SCD. Categories included limited self-care, limited provider knowledge on SCD, provider disrespect or dismissiveness, inadequate care, and the impact of disease on personal relationships. Limited provider knowledge on SCD is illustrated by the following quote, “They went to school and learned from books, but they can't know everything, you know” [LS3]. Many participants expressed that they were displeased by the responses from the providers when they asked questions about managing their disease. Participants also discussed the experience of feeling dismissed or disrespected by a provider, “You gonna get in and they ain’t going to listen to nothing you tell them” [LS1]. Participants also felt the care they received from the providers was inadequate, “It don't matter the disease, sickle cell, we still treated like some of the bottom” [LS3]. As indicated in Textbox 4, this treatment was often experienced in emergency room settings. In addition, the participants described the toll that the disease management took on their personal relationships. One participant stated, “I can take a big amount of pain, so I try to hide my pain because it’s people that depend on me and I don’t be wanting to let them down” [LS3]. Participants also shared that they “hide their pain because they do not want others to feel sorry for them.”

**Challenges of disease management. Illustrative quotes are examples from community listening participants recruited to develop the categories.**

**Illustrative quotes**

**Limited self-care:**

- “It's like I don't care how intense the pain is, I'll check and see how I feel tomorrow. I'll check tomorrow. If I put it off hopefully I’ll start feeling better.” [LS2]
- “Partying, drinking knowing that you can dehydrates us and put us in a crisis. Just doing everything that we shouldn't be doing.” [LS4]
- “If I don't take my medicine on a daily basis it because I don't feel good and I go through that blood issue a lot and I don't feel good.” [LS4]
- “I’m just stubborn you know? My wife has to make me go to the hospital.” [LS1]

**Limited provider knowledge on sickle cell disease:**

- “I could remember at [...] State, and being in the health center and they didn’t have a clue as to, you know, what to do.” [LS1]
- “Yeah, more than my doctors. They think they know everything.” [LS3]
- “What may work for me doesn't work for you. What may work for him, doesn't work for her. The doctors need to realize that.” [LS4]
- “When I was sick as a child. They had no idea what sickle cell was in the town that I grew up in.” [LS4]
- “Right now sickle cell is one page in their medical book.” [LS4]
- “I feel like I'm treated better when somebody is with me. when you go to the ER with them, the time that it takes for them to get you back, and get your information, and to see if it's okay to get medication, or IV.” [LS4]
- “Sickle cell, we still treated like some of the bottom. No matter the disease period, it has to start somewhere.” [LS4]
- “There's going to be ignorance towards the disease, you'll just so happen to get most of it and it’s frustrating.” [LS4]
- “Lack of education of providers around SCD.” [LS5]

**Provider disrespect or dismissiveness:**

- “Like you said we tell them it's a 10, and they're like ‘Are you sure?’ You know, you don't, they don't say ‘Are you sure it's a 10?’” [LS1]
- “And I've had nurses try to, you know, you know, stick me. And I tell them, you know, ‘It's gonna be hard.’ Oh no, and then they missed it.” [LS1]
- “I had an episode where I went to the hospital and he came in, listened to me, ask me what was going on, walked out the room, the nurse came back in and said, ‘He’s going to send you home.’ Didn't do nothing.” [LS4]
- “I can tell them, ‘This is what I need you to do, Do A, B, C, D.’ And guess what he does, D, F, G. He ain’t heard what I said. He ain’t heard nothing I just told him.” [LS4]
- “Because they get abused in the system. ‘Why are you here? Is this any different than before?’” [LS5]

**Inadequate care:**

- “But, if I get to the hospital, and they’re not giving me any more than I can already take at home, I’m getting frustrated.” [LS1]
- “You find it more on children but adult, you’re gonna be in the emergency room two or three hours before you get any help anyway and you might’ve could’ve stayed at home and got yourself together a little bit more, you know?” [LS2]
- “You gonna get in and they ain’t going to listen to nothing you tell them, so it’s like you was fighting a no win situation. [LS3]
- “I had a doctor walk in the room. I’m in full blown crisis. I’m sweating, everything going crazy. He walks in the room, look at me, “What’s wrong?” Did tell him what’s wrong. He walked right back out the room and never came back.” [LS4]
- “Even when we have therapy in general for pain management, we only have four slots a day for 260 patients, and that’s not guaranteed. That’s also a burden to kind of try to fix what we actually can.” [LS5]

**Impact of disease on personal relationships:**

- “Cause you go from spouse to mother.” [LS1]
- “With me, I can take a big amount of pain so I try to hide my pain because it's people that depend on me and I don't be wanting to let them down.” [LS3]

### Theme 5: Types of Support Received for Disease Management

This theme focuses on interpersonal relationships that can support maintaining a healthy lifestyle (Textbox 5). The categories of support that were discussed included emotional and informational support from family, health care accompaniment, and practical assistance with physical care. Participants explained the benefit of familial social support including, “I typically will call people that I know in my network, that understand the disease. Most of my friends, most of my family, people that I know, I've pretty much got them educated on it” [LS4]. This also suggests that SCD self-management may require patients to act as educators for members of their support network. The participants also discussed examples of families helping with physical care. For example, one participant stated, “People try to help with the massages and all” [LS3].

**Types of support received for disease management. Illustrative quotes are examples from community listening participants recruited to develop the categories.**

**Illustrative quotes**

**Familial:**

- “Helping me talk to the doctor. ‘Well what happen if she has to take this, this, and this?’” [LS1]
- “At this point it’s me and my husband understands. When I was younger it was my mom, my grandmother, my dad.” [LS4]
- “Yeah. Poor performing physicians get a lower salary reimbursement from the hospital, so they started paying attention to these families, but we have to empower the families to know how to complain.” [LS5]

**Health care accompaniment:**

- “Definitely nurse or advocate because my mom used to be a nurse at Vanderbilt, so. When it was her, she would go into nurse mode.” [LS1]
- “It's cold, I'm in pain and I'm mad and my boyfriends like, ‘Well what is this room? Where is she at on the list?’” [LS1]
- “He's still in the process of learning so when stuff happens, something like that, especially if we're going to a doctor... he can kind of answer the questions for me.” [LS1]
- “Never go by yourself you really can’t talk while you’re in pain. You’ll be sitting there.” [LS4]
- “The mother is not going home. The mother will say, Doc, this is what the national guidelines say. Why you sending me home?” [LS5]

**Physical care:**

- “Yeah, they know, you know, I’m supposed to take folic acid... supposed to take a mild [non-steroidal anti-inflammatory] if I feel any minor pain coming on.” [LS1]
- “Bring me some water or something or something.” [LS3]
- “People try to help with the massages and all.” [LS3]

### Theme 6: Barriers to and Facilitators of Using an mHealth App

This theme emphasizes the perceived obstacles and benefits of using an mHealth app to assist with disease management (Textbox 6). We identified 3 levels of barriers and facilitators: patient, app-specific, and provider. Patient-level barriers included characteristics of the participants that would prevent them from using the application. This included apprehension toward internet use. For example, “I do community outreach and a lot of people are not computer savvy. Some people don't want to have nothing to do with the Internet” [LS3]. In addition, participants acknowledged that after consistent use of the application, there was the potential to ignore alerts:

That takes to not turning your notifications off your phone. Cause I’m quick to turn the notification off on everything cause that phone....

LS4

The facilitators explained the benefits of using an mHealth app. These factors would make it easier for the participants to use or become more interested in using the app. For example, a participant reported, “so I think it would be very helpful, especially for a sister or a spouse, whoever is with you that day” [LS4]. Another participant shared that a facilitator to using an app was as follows:

...if it had simple interface, something easily configurable that I don’t have to go and do a lot...if it imports my medical records that would be fantastic.

LS1

**Barriers to and facilitators of using a mobile health app. Illustrative quotes are examples from community listening participants recruited to develop the categories.**

**Illustrative quotes**

**Patient level:**

- “Doesn’t pay the phone bill.” [LS1]
- “The same thing that would prevent me from forgetting my medicine. I would get busy, the change in schedule.” [LS2]
- “Some people are just not familiar with an app.” [LS3]
- “If you can't use internet, I don't know what would make it easier.” [LS3]
- “The problem is, she has had strokes so she can't do her hands in order to do things smartphone.” [LS3]
- “That takes to not turn your notifications off your phone. Cause I’m quick to turn the notification off on everything cause that phone get to bling, bling, bling, bling.” [LS4]
- “I probably open three of them because of practice and maybe one of them because of my own health.” [LS5]

**App-specific:**

- “If it’s not user-friendly you're not gonna get much success out of it either thought.” [LS1]
- “Some apps ask you to connect to different servers with both monitoring and notification and stuff like that.” [LS2-context]
- “A technology, especially in the world that we’re in and some stuff that we’re kind of working on already, has the ability because everybody has smart phones.” [LS5]

**Provider:**

- “Are the doctors buying into this app, because it’s just an app?” [LS4]
- “Each doctor going to do something different.” [LS4]
- “Okay, hypothetically, you go into the emergency room. And you show them this app. This is what I need and this is my information. And he don't follow it.” [LS4]

### Theme 7: Feature Preferences for an mHealth App

Feature preferences included various features that would facilitate SCD self-management. Four key features were identified: alert system, communication with providers, information tracker, and the ability to interact with caregivers (Textbox 7). Participants stated that they wanted an alert system. For example, “Alerts you or reminds you to...Don’t forget your folic acid” [LS1]. Another participant stated, “Put it on the phones so I can get a reminder” [LS4]. Participants wanted to be able to maintain communication with their providers through the mHealth app. One participant stated, “Even if the app was able to alert or connect to a patient registry or to allow them to email doctors if they are almost in a crisis” [LS2]. Other suggestions for an mHealth app were to have an information tracker and a feature that could enable an individual to interact with their caregiver.

**Feature preferences for a mobile health app. Illustrative quotes are examples from community listening participants recruited to develop the categories.**

**Illustrative quotes**

**Alert system:**

- “Alerts you or reminds you to...Don't forget your folic acid.” [LS1]
- “I would actually like for it to give me alerts or triggers.” [LS1]
- “With me, when I’m puttin’ in certain things like it tell you went over your carbs for the day or whatever put in the day it’s something that children realize until you get the alert.” [LS2]
- “Put it on the phones so I can get a reminder.” [LS4]

**Communication:**

- “Even if the app was able to alert or log in somehow connect to a patient registry or whatever to allow them to email doctors to alert them if they are almost in a crisis based on the information.” [LS2]
- “I think the app should have a daily tracker. Just somebody came how's your mood today? How are you feeling today?” [LS2]
- “Is it like a way the app could be linked into the hospital or clinic's computer cause that way any info you putting in the app already will be there anyway. I mean, if that’s the case then when you get to the emergency room or to the clinic... the computer already shows them whatever they need to know from your app.” [LS4]

**Information tracker:**

- “It shows like all your medications and the things you kind of track that stuff, track down the time that the nurses come in and give her medicine, what they giving her, how many milligrams.” [LS4]
- “I think the app should have a daily tracker. Just somebody came how’s your mood today? How are you feeling today? Over time I think that information would be helpful when you do have to go see your doctor...” [LS2]

**Ability to engage caregiver:**

- “Just preventative things to that a caretaker can read.” [LS1]
- “You tell the caregiver that, you know, make sure you go... Give them time for the medicine to work kind of thing.” [LS1]
- “Giving some expected measures of time for a caregiver to say, you know, ‘Hey check on them. Let them rest. You know, no noise, stress relief, close the door, shut the blinds.’” [LS1]
- “You can have like a preventive section for the caregiver to also refer to before it even gets to the point where you're at the hospital.” [LS1]
- “Leave me alone. Go look at the app.” [LS3]
- “I was going to ask from a caregiver’s perspective, we were talking about the app... an app where you kind of track that stuff, even stuff like, she’s a hard stick so there might be one person in the whole hospital that can get a line off her. Something simple like recording the name of that person.” [LS4]

## Discussion

### Principal Findings

Studies have demonstrated the efficacy of mHealth self-management interventions in improving clinical health outcomes. This study describes the preferences for mHealth app features to facilitate self-management in adults with SCD and their caregivers residing in urban and rural communities. The patient and caregiver inputs confirmed key findings from the literature and exposed the gaps in realizing the needs of patients with SCD and their caregivers in disease management.

In our study, participants were interested in using an SCD mHealth app to increase their ability to communicate with health care providers. This was similar to the findings of other qualitative studies on the benefits of mHealth for patients with chronic health conditions [37,38]. Furthermore, studies suggest that the most effective mHealth interventions for disease self-management connect patients with their health care team using 2-way communication [39,40]. Therefore, mHealth could be an effective method for meeting the patient communication needs. However, this communication was within the context of specialty care. One area that may not be as easily addressed through mHealth in SCD is communication in acute care settings.

The patients and caregivers in this study cited communication with providers in the emergency room as a challenge to disease management. In particular, they expressed concerns that they were not being heard and were often mistreated. These concerns are consistent with the health care experiences expressed among patients with SCD in other studies [41,42]. This reflects that although implementing mHealth in self-management interventions could potentially be effective in increasing communication with the health care providers, there is still a need to improve the SCD patient-provider interactions, particularly in acute settings.

Another major finding was the desire for patients to use mHealth to share SCD information with their caregivers. This was an expected finding, as participants also expressed that their main source of support for disease management was interpersonal support rather than a health care provider. Although few studies elicit preferences for mHealth among caregivers, obtaining feedback from them confirmed the benefit of adopting mHealth to facilitate communication between the patients and their caregivers [43,44]. Caregivers are often the main source of support for disease management in patients with chronic conditions, especially those residing in rural areas [45]. Health professionals or providers seeking to implement mHealth for expanding their services to rural patients should consider the usability of a proposed app for caregivers.

One consistent element in mHealth interventions is the use of reminder alerts or SMS text messaging [46,47]. Our study participants reported adhering to these alerts as a barrier to mHealth adoption. They stated that if they received several notifications from the app, they would shut off the notifications. This concern of alert fatigue, receiving a large quantity of information and having insufficient time or cognitive resources to distinguish relevant from irrelevant information [48,49], has also been described in provider-based health information technologies. As with this study’s participants, the result was overriding or dismissing alerts [50,51]. Therefore, health care professionals and providers seeking to implement these elements into an mHealth intervention should consider their potential barriers before implementation. The suggested strategies for overcoming this barrier can include allowing the users to prioritize alerts and to specify the types of alerts they would prefer receiving [50,51].

### Limitations

We acknowledge the limitations of this study. First, the community listening sessions included individuals with SCD and their caregivers. It is possible that responses may have varied if these 2 groups participated in separate listening sessions. Next, as with other qualitative approaches, the results are not generalizable outside the sample population. Finally, sampling bias may have affected the findings, as participants who were willing to participate in the community listening sessions may differ from the general population of patients with SCD and their caregivers. In particular, the caregivers in our study were parents and spouses of adults with SCD. Their perspectives may differ from those of caregivers who are the parents of children and adolescents with SCD. Despite these limitations, we believe that the contributions of this study outweigh the limitations. As technology is increasingly incorporated into SCD management, it is important to capture the perspectives of the 2 important groups most impacted, which are the patients and their caregivers.

### Conclusions

Patient and caregiver preferences are essential to understanding the components of mHealth technology that should be integrated into disease self-management to meet patient needs. This was one of the first studies to include the input from patients with SCD and their caregivers from rural communities. As technology-based strategies are continually developed to increase health care access in rural and medically underserved communities, it is important to incorporate the inputs from members of these communities. Future studies should assess the accessibility of technology resources available to patients with SCD residing in rural communities. These findings can be used to develop and enhance patient-centered, user-friendly mHealth apps that address the barriers to information access and facilitate disease self-management without creating an additional burden for the patients and their caregivers.

## Abbreviations

mHealth: mobile health
SCD: sickle cell disease
SCFT: Sickle Cell Foundation of Tennessee
